# Rate and affinity binding constants determined by SPR spectroscopy reveal differential antigenicity of HIV gp120 and gp140

**DOI:** 10.1186/1742-4690-9-S2-P59

**Published:** 2012-09-13

**Authors:** TL Gearhart, JD Steckbeck, JK Craigo, RC Montelaro

**Affiliations:** 1 University of PIttsburgh, Pittsburgh, PA, USA

## Background

The development of a practical, effective AIDS vaccine that can produce enduring broadly protective immunity to natural exposure by diverse strains of HIV-1 remains the ultimate goal for controlling the ongoing AIDS epidemic. The success of an effective AIDS vaccine will likely depend on designing immunogens that elicit broadly neutralizing antibodies to circulating HIV-1 strains, and it is important to thoroughly understand the mechanism of binding of HIV antigens and neutralizing antibodies. The current study was designed to determine differences in the specificity and quantitative properties of antibody binding to gp120 or gp140 envelope proteins derived from natural HIV isolates.

## Methods

We previously described novel assays of Env-specific serum antibodies elicited by animal lentiviral and HIV infections and immunizations that measure qualitative properties of avidity and conformational dependence in an ELISA format. However, recent data have emphasized limitations of this format. To provide a more sensitive, specific, and reproducible format for our antibody assays, we have recently developed novel procedures using SPR spectroscopy as measured in the Biacore system to characterize real time reaction kinetics (association/dissociation rates).

## Results

The assays conducted under physiological conditions (37°C, nondenaturing conditions, etc.) clearly indicate the potential of these kinetic measurements of antigen-antibody binding to provide novel data not achieved with standard assays. We have used these binding assays and a panel of well-characterized monoclonal antibodies both to linear and conformational binding domains to characterize differences in reactivity between variant gp120 and gp140 antigens as determined by kinetic rates and affinity of antibody binding. We have identified significant distinguishing differences in several binding characteristics of monoclonal antibody binding to gp120 vs gp140 antigens.

## Conclusion

These kinetic parameters offer novel insights into the fundamental interactions of reference monoclonal antibodies and variant Env proteins and have the potential to provide novel correlates of neutralization in vitro and protection in vivo.

